# Library as Safe Haven: Disaster Planning, Response, and Recovery

**DOI:** 10.5195/jmla.2017.335

**Published:** 2017-10-01

**Authors:** Eleanor Shanklin Truex

As of 2011, libraries are included in the Federal Emergency Management Agency’s (FEMA’s) designation as temporary relocation facilities during major disasters and emergencies. Part of the FEMA public assistance program, the designation makes disaster planning and effective emergency response more important than ever for all libraries.

This serviceable manual, *Library as Safe Haven: Disaster Planning, Response, and Recovery*, provides a common-sense overview of the methods used to prepare any library for a variety of worst case scenarios: “Utilizing a model that focuses on continuity of core resources and services while enhancing the library’s role through partnering with emergency planners, [this book] is designed primarily for libraries; however, the content and resources contained within can easily be adapted to any business or agency in search of a well-structured approach to readiness and disaster response” (p. ix). I would extend that applicability to personal property as well.

Being able to quickly extract pertinent advice and instruction from this manual is one of its strengths. The concise and clear graphics are another. When I first flipped through this book, the tables and checklists are what caught my eye. Their flexibility and comprehensiveness is outstanding, especially the “Vulnerability Table” on page 11. It sent this reviewer immediately into a quick, rough risk assessment of her libraries.

The templates are well organized and seem intended for group or committee use. The Service Continuity Pocket Response Plan (SCPReP), delineated on pages 47–58, is a vital resource for any library (or business) to have on hand. It is a template for a 1-page (double-sided) compendium of the essential contacts, resources, communication structure, library layout, and item rescue priority list compiled by the staff in the library as part of their disaster planning. Such a document is library recovery at-a-glance, intended to be convenient and accessible by library and nonlibrary personnel.

Along with the exemplary checklists, the index and the appendix for the book are comprehensive, providing another access route for quick information retrieval. While electronic resources can be as ephemeral as clouds, the provided list alerts readers to aspects of service recovery that they might not have considered. The coverage of social media’s usefulness, and mobile technology options (i.e., apps) is a highly useful addition, while the “old standby” of the print textbook list for a mobile disaster cart is a pertinent reminder that there will always be a place for current printed material in any library, if only for this sole purpose.

The text is clearly laid out and follows a sensible framework, starting with risk assessment, moving through threat response, self-reliance or external support, continuity planning, encouragement of the use of social media and mobile technology, personal preparedness, provision of support to rescuers and victims, and finally, two model scenarios that incorporate all of the above elements. Of note is the secondary effect of disaster striking library staff, but not the physical library plant. Inclusion of this scenario helps broaden the consideration of a disaster’s effects and is key to any comprehensive guide.

Disasters specifically covered include the “common”: hurricanes, earthquakes, tornadoes, floods, and other extreme weather. More unique disasters such as pandemics, active shooters, riots, and hazardous materials spills are also discussed, making this manual more comprehensive than other published offerings that this reviewer is aware of.

Looking at the many other books on this subject, Guy Robertson’s *Disaster Planning for Libraries: Process and Guidelines* (Chandos Publishing; 2015; ISBN: 978-1-843347309) is comparable in scope and resources. Robertson’s book does not present its information on the same level of detail as *Library as Safe Haven*, but it does include a “How to Use This Book” section that *Library as Safe Haven* could benefit from. It also presents the disasters of various pest invasions, which *Library as Safe Haven* omits, unfortunately. Robertson’s guide has a chattier tone and is a more entertaining read, but for solid concise information, this reviewer prefers *Library as Safe Haven*.

*Library as Safe Haven*’s focus is on public libraries, which is understandable. But there are many corporate or special libraries for which this guide is not quite complete. It is a weakness that this book does not cover those libraries that function as departments within larger institutions that may (and often do) have their own priorities for disaster planning and often do not include the library in the most essential of those, especially in the delegation of authority. This is a mistake on those institutions’ part. It is incumbent on librarians so employed to advocate for their place on any disaster or restoration planning committee. Still, it seems disingenuous that some of the disaster examples in the book draw from special libraries’ experiences, and yet the liaison between the library and the larger institution housing it is not explored. There may be aspects of this unique position that need to be addressed (corporate politics of departmental support, physical location in the institution, staffing, etc.) and that could be beneficial to the manual’s larger audience. This lapse, though, is not a deal-breaker for this comprehensive and extremely practical manual.

Every library should have a copy of this book, just as every library should have a disaster plan ready. Collections are too valuable and too expensive to replace easily; some prior effort along with maintenance of the accepted strategy can avert such losses. This book provides the roadmap for all libraries to survive “the worst” and continue to serve their patrons despite challenges created by nature or by humans.

